# 3β-Acet­oxy-5α-cholestan-6-one 2-cyano­acetyl­hydrazone

**DOI:** 10.1107/S1600536812001432

**Published:** 2012-01-21

**Authors:** Samina Khan Yusufzai, Hasnah Osman, Othman Sulaiman, Suhana Arshad, Ibrahim Abdul Razak

**Affiliations:** a School of Chemical Sciences, Universiti Sains Malaysia, 11800 USM, Penang, Malaysia; b School of Industrial Technology, Universiti Sains Malaysia, 11800 USM, Penang, Malaysia; c School of Physics, Universiti Sains Malaysia, 11800 USM, Penang, Malaysia

## Abstract

The asymmetric unit of the title compound, C_32_H_51_N_3_O_3_, consists of two crystallographically independent mol­ecules, *A* and *B*; the 2-methyl­pentane group of mol­ecule *A* and the propane group of mol­ecule *B* are each disordered over two sets of sites, with refined site-occupancies of 0.825 (5):0.175 (5) and 0.630 (18):0.370 (18), respectively. In both mol­ecules, the three cyclo­hexane rings in the steroid fused ring systems adopt chair conformations while the cyclo­pentane rings adopt envelope and twist conformations in mol­ecules *A* and *B*, respectively. In the crystal, N—H⋯O and C—H⋯O hydrogen bonds link the two independent mol­ecules together, generating *R*_2_^1^(7) and *R*_2_^2^(8) ring motifs.

## Related literature

For the biological activity of steroidal derivatives, see: Khan & Yusuf (2009 ▶); Drach *et al.* (2000 ▶); Gupta *et al.* (1995 ▶); Ahmed & Boruah (1996 ▶); Short & Long (1969 ▶); Khan *et al.* (2007 ▶); Doorenbos & Wu (1968 ▶); Banday *et al.* (2010 ▶). For ring conformations, see: Cremer & Pople (1975 ▶). For bond-length data, see: Allen *et al.* (1987 ▶). For related structures, see: Khan *et al.* (2011 ▶); Ketuly *et al.* (2011 ▶). For hydrogen-bond motifs, see: Bernstein *et al.* (1995 ▶). For stability of the temperature controller used in the data collection, see: Cosier & Glazer (1986 ▶).
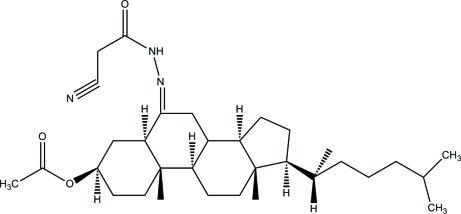


## Experimental

### 

#### Crystal data

C_32_H_51_N_3_O_3_
*M* *_r_* = 525.76
Monoclinic, 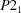

*a* = 17.1691 (2) Å
*b* = 9.6694 (1) Å
*c* = 19.1447 (3) Å
β = 109.171 (1)°
*V* = 3002.04 (7) Å^3^
*Z* = 4
Mo *K*α radiation
μ = 0.07 mm^−1^
*T* = 100 K
0.32 × 0.18 × 0.09 mm

#### Data collection

Bruker SMART APEXII CCD area-detector diffractometer
Absorption correction: multi-scan (*SADABS*; Bruker, 2009 ▶) *T*_min_ = 0.977, *T*_max_ = 0.994
40102 measured reflections
9291 independent reflections
6778 reflections with *I* > 2σ(*I*)
*R*_int_ = 0.081

#### Refinement

*R*[*F* ^2^ > 2σ(*F* ^2^)] = 0.054
*wR*(*F* ^2^) = 0.117
*S* = 1.01
9291 reflections
752 parameters
48 restraints
H atoms treated by a mixture of independent and constrained refinement
Δρ_max_ = 0.27 e Å^−3^
Δρ_min_ = −0.26 e Å^−3^

### 

Data collection: *APEX2* (Bruker, 2009 ▶); cell refinement: *SAINT* (Bruker, 2009 ▶); data reduction: *SAINT*; program(s) used to solve structure: *SHELXTL* (Sheldrick, 2008 ▶); program(s) used to refine structure: *SHELXTL*; molecular graphics: *SHELXTL*; software used to prepare material for publication: *SHELXTL* and *PLATON* (Spek, 2009 ▶).

## Supplementary Material

Crystal structure: contains datablock(s) global, I. DOI: 10.1107/S1600536812001432/is5021sup1.cif

Structure factors: contains datablock(s) I. DOI: 10.1107/S1600536812001432/is5021Isup2.hkl

Additional supplementary materials:  crystallographic information; 3D view; checkCIF report

## Figures and Tables

**Table 1:** Hydrogen-bond geometry (Å, °)

*D*—H⋯*A*	*D*—H	H⋯*A*	*D*⋯*A*	*D*—H⋯*A*
N2*A*—H1*NA*⋯O3*B*	0.85 (3)	2.09 (3)	2.932 (3)	170 (3)
N2*B*—H1*NB*⋯O3*A*	0.88 (3)	2.11 (3)	2.963 (3)	163 (3)
C1*A*—H1*AB*⋯O3*B*	0.97	2.39	3.201 (3)	140

**Fig. 1.**
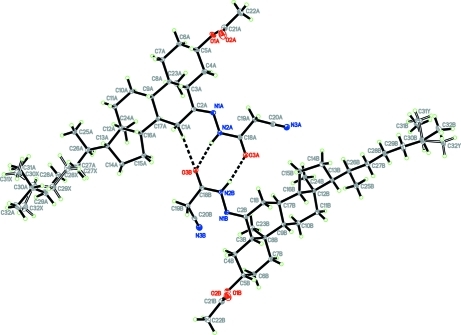
The molecular structure of the title compound, showing 30% probability displacement ellipsoids and the atom-numbering scheme. All disordered components are shown.

**Fig. 2.**
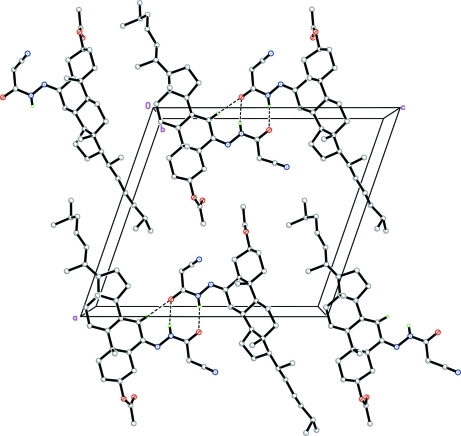
The crystal packing of the title compound. Only major disordered components is shown.

## supplementary crystallographic information

### Comment

A literature survey reveals that steroids are compounds of biological origin and play an important role in biological systems. During the last decade the major efforts of the chemists were directed towards the modification in the structure of steroids in order to enhance their biologically activity (Khan & Yusuf, 2009; Drach *et al.*, 2000). Many of these steroidal derivatives, were found to possess antimicrobial, anti-cancer, anti-inflammatory, anti-tuberculosis, hypotensive, anti-convulsant and diuretic activities (Gupta *et al.*, 1995; Ahmed & Boruah, 1996; Short & Long, 1969; Khan *et al.*, 2007; Doorenbos & Wu, 1968; Banday *et al.*, 2010). In the present work, we have synthesized a new compound, 3β-acetoxy-5α-cholestan-6-one-cyanoacetohydrazone which corresponds to the molecular formula C_32_H_51_N_3_O_3_. Synthesis of other derivatives of steroidal cyanoacetohydrazone and their biological activities are under progress.

The asymmetric unit of the title compound consists of two crystallographically independent molecules *A* and *B* (Fig. 1). The 2-methylpentane group of molecule *A* and the propane group of molecule *B* are disordered with refined site-occupancies of 0.825 (5): 0.175 (5) and 0.630 (18): 0.370 (18), respectively. For each molecules, the three cyclohexane rings in the steroid fused ring system adopt a chair conformation [Molecule *A* (C1A–C3A/C8A/C9A/C17A):(C3A–C8A): (C9A–C12A/C16A/C17A); Q= 0.545 (3):0.562 (3):0.569 (3) Å, Θ= 161.2 (3): 178.7 (3):176.4 (3)° and Φ= 21.3 (9):232 (10):24 (4)°; Molecule *B* (C1B–C3B/C8B/C9B/C17B):(C3B–C8B):(C9B–C12B/C16B/C17B); Q= 0.597 (3): 0.590 (3):0.593 (3) Å, Θ= 167.1 (3):172.9 (3):175.2 (3)° and Φ= 311.6 (12):294 (2):102 (3)°]. On the other hand, the cyclopentane ring of the steroid fused ring system in both molecules adopts a different ring conformation (Cremer & Pople, 1975). The cyclopentane (C12A–C16A) ring of molecule *A* is in an envelope conformation with puckering parameters Q= 0.469 (3) Å and φ= 351.6 (4)° with atom C12A at the flap. Meanwhile, molecule *B* is in a twist conformation where the cyclopentane (C12B–C16B) ring is twisted about C16B–C12B bonds, with puckering parameters Q= 0.464 (3) Å and φ= 350.2 (4)°. The bond lengths (Allen *et al.*, 1987) and angles are within normal ranges and are comparable to the related structures (Khan *et al.*, 2011; Ketuly *et al.*, 2011).

The crystal packing is shown in Fig. 2. Molecule *A* and *B* are interconnected *via* N2A—H1NA···O3B, N2B—H1NB···O3A and C1A—H1AB···O3B intermolecular hydrogen bonds (Table 1), generating *R*^1^_2_(7) and *R*^2^_2_(8) ring motifs (Bernstein *et al.*, 1995).

### Experimental

To a solution of steroidal ketone, 3β-acetoxy-5α-cholestan-6-one, (5 mmol) in absolute ethanol (10 ml) was added cyanoacetichydrazide (10 mmol) followed by few drops of triethylamine. The reaction mixture was refluxed for 24 h. The progress of reaction was monitored by thin layer chromatography. After completion of the reaction, reaction mixture was concentrated under reduce pressure. The obtained solid, was extracted with ether and ethereal layer was washed with water, NaHCO_3_ solution (5%) and dried over anhydrous sodium sulfate. The solvent was evaporated and the product was recrystallized from ethanol resulting in shiny crystals.

### Refinement

The propane group of molecule *A* was disordered over two positions with refined site-occupancies of 0.825 (5):0.175 (5). The minor disordered components were refined isotropically. For molecule *B*, the 2-methylpentane group was disordered over two positions with refined site-occupancies of 0.630 (18):0.370 (18). N-bound H atoms was located from the difference map and refined freely, [N—H = 0.85 (3) and 0.88 (3) Å]. The remaining H atoms were positioned geometrically [C—H = 0.96–0.98 Å] and refined using a riding model with *U*_iso_(H) = 1.2 or 1.5 *U*_eq_(C). A rotating group model was applied to the methyl groups. Since there are not sufficient anomalous dispersion to determine the absolute configuration, 8296 Friedel pairs were merged for the final refinement. The same *U^ij^* parameter was used for atoms pair C30X/C31X. Similarity and rigid bond restraints was also used in the final refinement.

### Crystal data

**Table:**

C_32_H_51_N_3_O_3_	*F*(000) = 1152
*M**_r_* = 525.76	*D*_x_ = 1.163 Mg m^−^^3^
Monoclinic, *P*2_1_	Mo *K*α radiation, λ = 0.71073 Å
Hall symbol: P 2yb	Cell parameters from 5129 reflections
*a* = 17.1691 (2) Å	θ = 2.9–30.1°
*b* = 9.6694 (1) Å	µ = 0.07 mm^−^^1^
*c* = 19.1447 (3) Å	*T* = 100 K
β = 109.171 (1)°	Plate, colourless
*V* = 3002.04 (7) Å^3^	0.32 × 0.18 × 0.09 mm
*Z* = 4	

### Data collection

**Table:**

Bruker SMART APEXII CCD area-detector diffractometer	9291 independent reflections
Radiation source: fine-focus sealed tube	6778 reflections with *I* > 2σ(*I*)
graphite	*R*_int_ = 0.081
φ and ω scans	θ_max_ = 30.1°, θ_min_ = 2.4°
Absorption correction: multi-scan (*SADABS*; Bruker, 2009)	*h* = −24→24
*T*_min_ = 0.977, *T*_max_ = 0.994	*k* = −13→13
40102 measured reflections	*l* = −26→26

### Refinement

**Table:**

Refinement on *F*^2^	Primary atom site location: structure-invariant direct methods
Least-squares matrix: full	Secondary atom site location: difference Fourier map
*R*[*F*^2^ > 2σ(*F*^2^)] = 0.054	Hydrogen site location: inferred from neighbouring sites
*wR*(*F*^2^) = 0.117	H atoms treated by a mixture of independent and constrained refinement
*S* = 1.01	*w* = 1/[σ^2^(*F*_o_^2^) + (0.0539*P*)^2^ + 0.0496*P*] where *P* = (*F*_o_^2^ + 2*F*_c_^2^)/3
9291 reflections	(Δ/σ)_max_ = 0.001
752 parameters	Δρ_max_ = 0.27 e Å^−^^3^
48 restraints	Δρ_min_ = −0.26 e Å^−^^3^

### Special details

**Table:**

Experimental. The crystal was placed in the cold stream of an Oxford Cryosystems Cobra open-flow nitrogen cryostat (Cosier & Glazer, 1986) operating at 100.0 (1) K.
Geometry. All e.s.d.'s (except the e.s.d. in the dihedral angle between two l.s. planes) are estimated using the full covariance matrix. The cell e.s.d.'s are taken into account individually in the estimation of e.s.d.'s in distances, angles and torsion angles; correlations between e.s.d.'s in cell parameters are only used when they are defined by crystal symmetry. An approximate (isotropic) treatment of cell e.s.d.'s is used for estimating e.s.d.'s involving l.s. planes.
Refinement. Refinement of *F*^2^ against ALL reflections. The weighted *R*-factor *wR* and goodness of fit *S* are based on *F*^2^, conventional *R*-factors *R* are based on *F*, with *F* set to zero for negative *F*^2^. The threshold expression of *F*^2^ > σ(*F*^2^) is used only for calculating *R*-factors(gt) *etc*. and is not relevant to the choice of reflections for refinement. *R*-factors based on *F*^2^ are statistically about twice as large as those based on *F*, and *R*- factors based on ALL data will be even larger.

### Fractional atomic coordinates and isotropic or equivalent isotropic displacement parameters (Å^2^)

**Table:**

	*x*	*y*	*z*	*U*_iso_*/*U*_eq_	Occ. (<1)
O1A	0.57377 (11)	0.8338 (2)	0.71592 (11)	0.0267 (5)	
O2A	0.56068 (14)	1.0550 (3)	0.67883 (13)	0.0452 (6)	
O3A	0.90699 (10)	0.7992 (2)	0.49807 (10)	0.0247 (4)	
N1A	0.83483 (12)	0.7956 (2)	0.64950 (11)	0.0172 (4)	
N2A	0.88885 (13)	0.7881 (2)	0.60893 (12)	0.0181 (4)	
N3A	0.72058 (15)	0.6629 (3)	0.37315 (14)	0.0329 (6)	
C1A	0.95621 (14)	0.7990 (3)	0.76523 (14)	0.0209 (6)	
H1AA	0.9759	0.8937	0.7726	0.025*	
H1AB	0.9857	0.7523	0.7366	0.025*	
C2A	0.86589 (14)	0.8009 (3)	0.72046 (14)	0.0164 (5)	
C3A	0.80581 (15)	0.8246 (3)	0.76226 (13)	0.0175 (5)	
H3AA	0.8148	0.9201	0.7801	0.021*	
C4A	0.71486 (15)	0.8168 (3)	0.71406 (15)	0.0212 (5)	
H4AA	0.7047	0.8794	0.6724	0.025*	
H4AB	0.7018	0.7237	0.6949	0.025*	
C5A	0.66004 (15)	0.8559 (3)	0.75950 (15)	0.0230 (6)	
H5AA	0.6687	0.9534	0.7740	0.028*	
C6A	0.67590 (15)	0.7673 (3)	0.82741 (15)	0.0270 (6)	
H6AA	0.6600	0.6726	0.8127	0.032*	
H6AB	0.6426	0.8000	0.8563	0.032*	
C7A	0.76765 (15)	0.7717 (3)	0.87501 (14)	0.0241 (6)	
H7AA	0.7809	0.8642	0.8950	0.029*	
H7AB	0.7765	0.7087	0.9163	0.029*	
C8A	0.82676 (15)	0.7330 (3)	0.83276 (14)	0.0167 (5)	
C9A	0.91689 (14)	0.7667 (3)	0.88116 (13)	0.0156 (5)	
H9AA	0.9197	0.8675	0.8867	0.019*	
C10A	0.94319 (15)	0.7075 (3)	0.95982 (13)	0.0197 (5)	
H10A	0.9376	0.6076	0.9570	0.024*	
H10B	0.9065	0.7425	0.9849	0.024*	
C11A	1.03259 (14)	0.7448 (3)	1.00555 (14)	0.0201 (5)	
H11A	1.0366	0.8438	1.0139	0.024*	
H11B	1.0470	0.6995	1.0533	0.024*	
C12A	1.09402 (15)	0.7013 (3)	0.96700 (13)	0.0169 (5)	
C13A	1.18305 (14)	0.7622 (3)	0.99766 (14)	0.0192 (5)	
H13A	1.1777	0.8607	1.0073	0.023*	
C14A	1.21409 (15)	0.7522 (3)	0.93005 (13)	0.0195 (5)	
H14A	1.2468	0.8329	0.9279	0.023*	
H14B	1.2479	0.6704	0.9339	0.023*	
C15A	1.13686 (14)	0.7442 (3)	0.85991 (14)	0.0198 (5)	
H15A	1.1384	0.8150	0.8245	0.024*	
H15B	1.1325	0.6542	0.8366	0.024*	
C16A	1.06532 (14)	0.7688 (3)	0.88967 (13)	0.0166 (5)	
H16A	1.0639	0.8686	0.8980	0.020*	
C17A	0.97830 (14)	0.7285 (3)	0.84107 (13)	0.0165 (5)	
H17A	0.9766	0.6281	0.8337	0.020*	
C18A	0.85976 (14)	0.7953 (3)	0.53426 (14)	0.0183 (5)	
C19A	0.76648 (15)	0.8000 (3)	0.49751 (14)	0.0213 (5)	
H19A	0.7400	0.7593	0.5303	0.026*	
H19B	0.7486	0.8954	0.4886	0.026*	
C20A	0.74160 (16)	0.7241 (3)	0.42715 (15)	0.0229 (6)	
C21A	0.53096 (18)	0.9421 (4)	0.67993 (17)	0.0306 (7)	
C22A	0.44342 (17)	0.9025 (4)	0.63900 (18)	0.0406 (8)	
H22A	0.4109	0.9845	0.6233	0.061*	
H22B	0.4218	0.8496	0.6710	0.061*	
H22C	0.4414	0.8480	0.5965	0.061*	
C23A	0.81746 (17)	0.5795 (3)	0.81186 (16)	0.0235 (6)	
H23A	0.8394	0.5243	0.8556	0.035*	
H23B	0.8470	0.5600	0.7782	0.035*	
H23C	0.7601	0.5581	0.7886	0.035*	
C24A	1.09831 (16)	0.5431 (3)	0.96309 (15)	0.0220 (6)	
H24A	1.0468	0.5082	0.9307	0.033*	
H24B	1.1090	0.5049	1.0116	0.033*	
H24C	1.1418	0.5173	0.9445	0.033*	
C25A	1.21170 (17)	0.6917 (3)	1.13305 (14)	0.0248 (6)	
H25A	1.1682	0.6244	1.1223	0.037*	
H25B	1.1906	0.7804	1.1405	0.037*	
H25C	1.2553	0.6650	1.1770	0.037*	
C26A	1.24532 (16)	0.7000 (3)	1.06800 (14)	0.0242 (6)	
H26B	1.2595	0.6064	1.0566	0.029*	0.825 (5)
H26C	1.2449	0.6024	1.0541	0.029*	0.175 (5)
C27A	1.3267 (2)	0.7912 (5)	1.0919 (2)	0.0233 (8)	0.825 (5)
H27A	1.3423	0.8106	1.0486	0.028*	0.825 (5)
H27B	1.3152	0.8788	1.1113	0.028*	0.825 (5)
C28A	1.3991 (2)	0.7212 (4)	1.1505 (2)	0.0314 (9)	0.825 (5)
H28A	1.3828	0.7007	1.1933	0.038*	0.825 (5)
H28B	1.4104	0.6338	1.1307	0.038*	0.825 (5)
C29A	1.4787 (2)	0.8059 (4)	1.1757 (2)	0.0314 (9)	0.825 (5)
H29A	1.5209	0.7511	1.2110	0.038*	0.825 (5)
H29B	1.4965	0.8215	1.1333	0.038*	0.825 (5)
C30A	1.47384 (19)	0.9447 (4)	1.2109 (2)	0.0253 (8)	0.825 (5)
H30A	1.4389	1.0063	1.1727	0.030*	0.825 (5)
C31A	1.4363 (3)	0.9328 (5)	1.2731 (3)	0.0296 (10)	0.825 (5)
H31A	1.3800	0.9034	1.2529	0.044*	0.825 (5)
H31B	1.4386	1.0211	1.2966	0.044*	0.825 (5)
H31C	1.4669	0.8663	1.3089	0.044*	0.825 (5)
C32A	1.5599 (2)	1.0087 (5)	1.2413 (3)	0.0410 (11)	0.825 (5)
H32A	1.5837	1.0149	1.2025	0.062*	0.825 (5)
H32B	1.5942	0.9518	1.2805	0.062*	0.825 (5)
H32C	1.5559	1.0996	1.2600	0.062*	0.825 (5)
C27X	1.3244 (12)	0.728 (2)	1.0891 (12)	0.025 (5)*	0.175 (5)
H27C	1.3461	0.7083	1.0493	0.030*	0.175 (5)
H27D	1.3538	0.6716	1.1313	0.030*	0.175 (5)
C28X	1.3364 (11)	0.880 (2)	1.1096 (11)	0.032 (4)*	0.175 (5)
H28C	1.3094	0.9006	1.1456	0.038*	0.175 (5)
H28D	1.3104	0.9355	1.0659	0.038*	0.175 (5)
C29X	1.4293 (9)	0.9195 (18)	1.1422 (9)	0.028 (4)*	0.175 (5)
H29C	1.4568	0.8890	1.1080	0.033*	0.175 (5)
H29F	1.4335	1.0194	1.1453	0.033*	0.175 (5)
C30X	1.4744 (9)	0.860 (2)	1.2173 (11)	0.036 (4)*	0.175 (5)
H30B	1.4566	0.7658	1.2228	0.043*	0.175 (5)
C31X	1.4547 (17)	0.961 (3)	1.2710 (16)	0.036 (4)*	0.175 (5)
H31D	1.4903	0.9430	1.3204	0.054*	0.175 (5)
H31E	1.3983	0.9500	1.2685	0.054*	0.175 (5)
H31F	1.4632	1.0545	1.2576	0.054*	0.175 (5)
C32X	1.5679 (10)	0.870 (3)	1.2335 (13)	0.052 (6)*	0.175 (5)
H32D	1.5825	0.8215	1.1958	0.077*	0.175 (5)
H32E	1.5959	0.8285	1.2807	0.077*	0.175 (5)
H32F	1.5838	0.9650	1.2342	0.077*	0.175 (5)
O1B	1.40344 (10)	0.9093 (2)	0.44593 (10)	0.0257 (4)	
O2B	1.39603 (14)	1.1422 (3)	0.44671 (13)	0.0406 (6)	
O3B	1.06940 (10)	0.8041 (2)	0.66162 (10)	0.0235 (4)	
N1B	1.13933 (12)	0.8916 (2)	0.51634 (12)	0.0179 (4)	
N2B	1.08365 (13)	0.8767 (2)	0.55456 (12)	0.0191 (5)	
N3B	1.27080 (15)	0.6266 (3)	0.60085 (14)	0.0314 (6)	
C1B	1.02418 (14)	0.9475 (3)	0.39848 (14)	0.0174 (5)	
H1BA	1.0208	1.0364	0.3742	0.021*	
H1BB	0.9883	0.9500	0.4284	0.021*	
C2B	1.11173 (14)	0.9215 (3)	0.44725 (14)	0.0160 (5)	
C3B	1.17228 (14)	0.9097 (3)	0.40589 (13)	0.0156 (5)	
H3BA	1.1624	0.9882	0.3717	0.019*	
C4B	1.26343 (14)	0.9150 (3)	0.45409 (14)	0.0194 (5)	
H4BA	1.2753	1.0016	0.4810	0.023*	
H4BB	1.2759	0.8397	0.4896	0.023*	
C5B	1.31564 (14)	0.9021 (3)	0.40366 (14)	0.0200 (5)	
H5BA	1.3014	0.9769	0.3671	0.024*	
C6B	1.30073 (15)	0.7643 (3)	0.36409 (15)	0.0220 (6)	
H6BA	1.3136	0.6896	0.3999	0.026*	
H6BB	1.3360	0.7555	0.3338	0.026*	
C7B	1.20998 (14)	0.7558 (3)	0.31530 (14)	0.0188 (5)	
H7BA	1.2002	0.6664	0.2910	0.023*	
H7BB	1.1995	0.8260	0.2771	0.023*	
C8B	1.14854 (14)	0.7754 (3)	0.35791 (13)	0.0161 (5)	
C9B	1.06048 (14)	0.7990 (3)	0.30241 (13)	0.0167 (5)	
H9BA	1.0639	0.8809	0.2734	0.020*	
C10B	1.02858 (15)	0.6814 (3)	0.24687 (14)	0.0215 (6)	
H10C	1.0236	0.5983	0.2735	0.026*	
H10D	1.0687	0.6632	0.2222	0.026*	
C11B	0.94502 (15)	0.7126 (3)	0.18838 (14)	0.0207 (5)	
H11C	0.9267	0.6318	0.1573	0.025*	
H11D	0.9515	0.7879	0.1573	0.025*	
C12B	0.87914 (14)	0.7520 (3)	0.22299 (13)	0.0170 (5)	
C13B	0.80126 (15)	0.8260 (3)	0.17172 (14)	0.0184 (5)	
H13B	0.8206	0.8945	0.1434	0.022*	
C14B	0.76889 (16)	0.9089 (3)	0.22640 (15)	0.0239 (6)	
H14C	0.7488	0.9989	0.2059	0.029*	
H14D	0.7241	0.8594	0.2357	0.029*	
C15B	0.84253 (15)	0.9252 (3)	0.29900 (14)	0.0207 (5)	
H15C	0.8506	1.0214	0.3141	0.025*	
H15D	0.8341	0.8712	0.3386	0.025*	
C16B	0.91555 (15)	0.8707 (3)	0.27870 (14)	0.0165 (5)	
H16B	0.9301	0.9445	0.2501	0.020*	
C17B	0.99553 (14)	0.8327 (3)	0.33988 (13)	0.0161 (5)	
H17B	0.9856	0.7487	0.3644	0.019*	
C18B	1.11377 (15)	0.8330 (3)	0.62498 (14)	0.0190 (5)	
C19B	1.20771 (15)	0.8238 (3)	0.65905 (14)	0.0224 (6)	
H19C	1.2317	0.9118	0.6527	0.027*	
H19D	1.2222	0.8066	0.7117	0.027*	
C20B	1.24303 (15)	0.7139 (3)	0.62561 (15)	0.0227 (6)	
C21B	1.43596 (18)	1.0379 (4)	0.46018 (16)	0.0311 (7)	
C22B	1.52840 (17)	1.0310 (5)	0.49423 (19)	0.0457 (9)	
H22D	1.5461	1.0919	0.5361	0.069*	
H22E	1.5534	1.0587	0.4584	0.069*	
H22F	1.5446	0.9380	0.5099	0.069*	
C23B	1.15183 (16)	0.6469 (3)	0.40651 (15)	0.0205 (5)	
H23D	1.1480	0.5648	0.3774	0.031*	
H23E	1.1066	0.6496	0.4255	0.031*	
H23F	1.2029	0.6463	0.4469	0.031*	
C24B	0.85473 (16)	0.6264 (3)	0.26037 (15)	0.0223 (6)	
H24D	0.8316	0.5561	0.2240	0.033*	
H24E	0.8147	0.6540	0.2828	0.033*	
H24F	0.9027	0.5906	0.2977	0.033*	
C25B	0.76610 (17)	0.6444 (3)	0.06897 (16)	0.0282 (6)	
H25D	0.7211	0.5976	0.0333	0.042*	
H25E	0.8019	0.5776	0.1009	0.042*	
H25F	0.7964	0.6965	0.0438	0.042*	
C26B	0.73254 (15)	0.7424 (3)	0.11504 (14)	0.0207 (5)	
H26A	0.7043	0.6868	0.1422	0.025*	
C27B	0.66974 (17)	0.8419 (3)	0.06442 (15)	0.0275 (6)	
H27E	0.6501	0.9030	0.0952	0.033*	
H27F	0.6981	0.8987	0.0386	0.033*	
C28B	0.59475 (16)	0.7767 (3)	0.00684 (15)	0.0266 (6)	
H28E	0.6135	0.7164	−0.0249	0.032*	
H28F	0.5655	0.7203	0.0320	0.032*	
C29B	0.5358 (2)	0.8828 (4)	−0.04063 (18)	0.0420 (8)	
H29D	0.5679	0.9517	−0.0561	0.050*	
H29E	0.5085	0.9294	−0.0102	0.050*	
C30B	0.46996 (18)	0.8271 (4)	−0.10934 (17)	0.0366 (8)	
H30C	0.4962	0.7974	−0.1452	0.044*	0.630 (18)
H30D	0.4958	0.7573	−0.1317	0.044*	0.370 (18)
C31B	0.4227 (4)	0.7039 (9)	−0.0914 (5)	0.0376 (18)	0.630 (18)
H31G	0.4582	0.6246	−0.0792	0.056*	0.630 (18)
H31H	0.3754	0.6834	−0.1337	0.056*	0.630 (18)
H31I	0.4050	0.7272	−0.0502	0.056*	0.630 (18)
C32B	0.4050 (5)	0.9398 (7)	−0.1451 (5)	0.047 (2)	0.630 (18)
H32G	0.3718	0.9566	−0.1142	0.071*	0.630 (18)
H32H	0.3704	0.9090	−0.1929	0.071*	0.630 (18)
H32I	0.4325	1.0237	−0.1504	0.071*	0.630 (18)
C31Y	0.4060 (8)	0.757 (2)	−0.0858 (9)	0.058 (4)	0.370 (18)
H31J	0.4293	0.6769	−0.0571	0.087*	0.370 (18)
H31K	0.3611	0.7298	−0.1287	0.087*	0.370 (18)
H31L	0.3861	0.8195	−0.0565	0.087*	0.370 (18)
C32Y	0.4369 (10)	0.9435 (11)	−0.1666 (6)	0.046 (3)	0.370 (18)
H32J	0.4812	0.9801	−0.1811	0.069*	0.370 (18)
H32K	0.4139	1.0158	−0.1450	0.069*	0.370 (18)
H32L	0.3950	0.9070	−0.2092	0.069*	0.370 (18)
H1NA	0.9409 (19)	0.794 (4)	0.6297 (18)	0.033 (9)*	
H1NB	1.0301 (17)	0.870 (3)	0.5324 (15)	0.021 (8)*	

### Atomic displacement parameters (Å^2^)

**Table:**

	*U*^11^	*U*^22^	*U*^33^	*U*^12^	*U*^13^	*U*^23^
O1A	0.0151 (8)	0.0312 (12)	0.0318 (11)	−0.0025 (8)	0.0047 (8)	−0.0029 (9)
O2A	0.0431 (13)	0.0400 (15)	0.0440 (14)	−0.0065 (11)	0.0025 (12)	0.0057 (12)
O3A	0.0203 (9)	0.0361 (12)	0.0181 (9)	−0.0048 (9)	0.0068 (8)	−0.0006 (9)
N1A	0.0180 (10)	0.0165 (11)	0.0186 (10)	−0.0001 (9)	0.0080 (9)	0.0010 (9)
N2A	0.0147 (10)	0.0229 (12)	0.0162 (10)	0.0020 (9)	0.0044 (9)	0.0021 (9)
N3A	0.0277 (13)	0.0465 (17)	0.0246 (13)	−0.0109 (12)	0.0087 (11)	−0.0060 (12)
C1A	0.0172 (11)	0.0268 (15)	0.0193 (12)	−0.0016 (11)	0.0067 (10)	0.0043 (12)
C2A	0.0177 (11)	0.0134 (12)	0.0197 (12)	−0.0006 (10)	0.0083 (10)	0.0004 (10)
C3A	0.0197 (12)	0.0169 (13)	0.0173 (12)	−0.0024 (10)	0.0079 (10)	−0.0029 (10)
C4A	0.0182 (11)	0.0226 (14)	0.0236 (13)	−0.0022 (11)	0.0082 (11)	−0.0015 (12)
C5A	0.0152 (11)	0.0277 (15)	0.0261 (14)	−0.0041 (11)	0.0066 (11)	−0.0044 (12)
C6A	0.0181 (12)	0.0397 (18)	0.0266 (14)	−0.0045 (12)	0.0121 (11)	−0.0047 (13)
C7A	0.0195 (12)	0.0360 (17)	0.0182 (12)	−0.0028 (12)	0.0080 (11)	−0.0022 (12)
C8A	0.0172 (11)	0.0188 (13)	0.0154 (11)	−0.0013 (10)	0.0071 (10)	−0.0018 (10)
C9A	0.0182 (11)	0.0141 (13)	0.0155 (11)	−0.0010 (10)	0.0066 (10)	0.0008 (10)
C10A	0.0199 (12)	0.0248 (14)	0.0167 (12)	−0.0004 (11)	0.0089 (10)	0.0019 (11)
C11A	0.0212 (12)	0.0220 (14)	0.0174 (12)	0.0036 (11)	0.0069 (10)	0.0002 (11)
C12A	0.0205 (12)	0.0190 (13)	0.0129 (11)	0.0012 (10)	0.0080 (10)	0.0000 (10)
C13A	0.0169 (11)	0.0231 (14)	0.0164 (12)	0.0026 (10)	0.0039 (10)	−0.0004 (11)
C14A	0.0197 (12)	0.0229 (14)	0.0170 (12)	0.0025 (10)	0.0075 (10)	0.0019 (11)
C15A	0.0186 (12)	0.0249 (14)	0.0178 (12)	−0.0010 (11)	0.0085 (10)	0.0024 (11)
C16A	0.0157 (11)	0.0171 (13)	0.0173 (12)	−0.0006 (10)	0.0058 (10)	0.0004 (10)
C17A	0.0201 (12)	0.0154 (13)	0.0152 (11)	0.0005 (10)	0.0075 (10)	0.0021 (10)
C18A	0.0168 (11)	0.0181 (13)	0.0187 (12)	−0.0032 (10)	0.0041 (10)	0.0000 (11)
C19A	0.0178 (11)	0.0285 (15)	0.0158 (12)	−0.0018 (11)	0.0030 (10)	−0.0011 (12)
C20A	0.0205 (12)	0.0287 (16)	0.0199 (13)	−0.0048 (11)	0.0073 (11)	0.0012 (12)
C21A	0.0272 (15)	0.0384 (19)	0.0252 (15)	−0.0001 (14)	0.0071 (13)	0.0005 (14)
C22A	0.0245 (14)	0.055 (2)	0.0349 (17)	−0.0022 (15)	0.0001 (14)	0.0073 (17)
C23A	0.0253 (13)	0.0203 (14)	0.0234 (14)	−0.0062 (11)	0.0059 (12)	0.0012 (11)
C24A	0.0249 (13)	0.0212 (14)	0.0212 (13)	0.0027 (11)	0.0091 (11)	0.0048 (11)
C25A	0.0260 (13)	0.0307 (16)	0.0165 (12)	0.0054 (12)	0.0055 (11)	−0.0010 (12)
C26A	0.0251 (13)	0.0298 (15)	0.0168 (12)	0.0075 (12)	0.0056 (11)	0.0006 (12)
C27A	0.0205 (16)	0.027 (3)	0.0222 (18)	0.0008 (16)	0.0065 (13)	−0.0025 (18)
C28A	0.0248 (17)	0.033 (2)	0.0283 (18)	0.0029 (15)	−0.0024 (15)	−0.0046 (17)
C29A	0.0191 (15)	0.043 (2)	0.0286 (18)	0.0039 (16)	0.0026 (14)	−0.0092 (18)
C30A	0.0147 (15)	0.030 (2)	0.0276 (19)	−0.0003 (14)	0.0027 (14)	0.0022 (16)
C31A	0.033 (2)	0.036 (3)	0.0245 (19)	−0.0042 (19)	0.0158 (18)	−0.0008 (18)
C32A	0.0228 (19)	0.045 (3)	0.050 (3)	−0.0071 (17)	0.0039 (19)	−0.006 (2)
O1B	0.0156 (8)	0.0364 (12)	0.0234 (10)	−0.0023 (8)	0.0040 (8)	−0.0004 (9)
O2B	0.0367 (12)	0.0372 (14)	0.0402 (13)	−0.0090 (11)	0.0021 (10)	0.0019 (12)
O3B	0.0203 (9)	0.0351 (11)	0.0161 (9)	−0.0058 (8)	0.0073 (7)	0.0002 (9)
N1B	0.0170 (10)	0.0216 (12)	0.0166 (10)	−0.0003 (9)	0.0078 (9)	−0.0024 (9)
N2B	0.0143 (10)	0.0299 (13)	0.0140 (10)	0.0007 (9)	0.0059 (9)	−0.0004 (9)
N3B	0.0278 (13)	0.0344 (15)	0.0303 (13)	0.0050 (11)	0.0072 (11)	0.0059 (12)
C1B	0.0175 (12)	0.0191 (13)	0.0168 (12)	0.0024 (10)	0.0073 (10)	−0.0013 (11)
C2B	0.0139 (11)	0.0148 (13)	0.0181 (12)	−0.0003 (10)	0.0037 (10)	−0.0018 (10)
C3B	0.0166 (11)	0.0168 (13)	0.0137 (11)	0.0013 (10)	0.0052 (10)	0.0019 (10)
C4B	0.0194 (12)	0.0218 (14)	0.0177 (12)	−0.0009 (11)	0.0069 (10)	0.0003 (11)
C5B	0.0147 (11)	0.0268 (15)	0.0180 (12)	0.0014 (11)	0.0045 (10)	0.0023 (11)
C6B	0.0186 (12)	0.0290 (15)	0.0201 (12)	0.0024 (11)	0.0087 (10)	−0.0009 (12)
C7B	0.0187 (11)	0.0215 (14)	0.0174 (12)	0.0033 (10)	0.0077 (10)	0.0001 (11)
C8B	0.0173 (11)	0.0173 (13)	0.0144 (11)	0.0020 (10)	0.0060 (10)	0.0002 (10)
C9B	0.0179 (11)	0.0166 (13)	0.0163 (11)	0.0027 (10)	0.0066 (10)	0.0011 (10)
C10B	0.0207 (13)	0.0241 (15)	0.0203 (13)	0.0013 (11)	0.0078 (11)	−0.0034 (11)
C11B	0.0213 (12)	0.0245 (14)	0.0166 (12)	−0.0007 (11)	0.0066 (10)	−0.0045 (11)
C12B	0.0173 (11)	0.0186 (13)	0.0151 (11)	0.0010 (10)	0.0055 (10)	0.0013 (10)
C13B	0.0177 (11)	0.0205 (14)	0.0162 (12)	−0.0009 (10)	0.0043 (10)	0.0026 (11)
C14B	0.0216 (12)	0.0268 (15)	0.0224 (13)	0.0016 (11)	0.0058 (11)	−0.0019 (12)
C15B	0.0198 (12)	0.0230 (14)	0.0176 (12)	0.0022 (11)	0.0039 (10)	−0.0034 (11)
C16B	0.0174 (11)	0.0175 (13)	0.0151 (11)	0.0019 (10)	0.0062 (10)	0.0020 (10)
C17B	0.0172 (11)	0.0159 (13)	0.0152 (11)	−0.0007 (10)	0.0051 (10)	0.0007 (10)
C18B	0.0178 (11)	0.0217 (14)	0.0179 (12)	−0.0033 (10)	0.0065 (10)	−0.0028 (11)
C19B	0.0176 (12)	0.0314 (16)	0.0169 (12)	−0.0016 (11)	0.0039 (10)	0.0000 (12)
C20B	0.0161 (12)	0.0283 (16)	0.0226 (13)	−0.0009 (11)	0.0048 (11)	0.0060 (12)
C21B	0.0245 (14)	0.045 (2)	0.0233 (15)	−0.0070 (14)	0.0071 (12)	0.0002 (15)
C22B	0.0243 (15)	0.072 (3)	0.0375 (19)	−0.0131 (17)	0.0057 (15)	−0.0032 (19)
C23B	0.0226 (13)	0.0163 (13)	0.0207 (13)	0.0013 (11)	0.0045 (11)	−0.0005 (11)
C24B	0.0252 (13)	0.0209 (14)	0.0193 (13)	−0.0020 (11)	0.0052 (11)	0.0027 (11)
C25B	0.0274 (14)	0.0322 (17)	0.0218 (14)	−0.0024 (13)	0.0037 (12)	−0.0058 (13)
C26B	0.0212 (12)	0.0223 (14)	0.0172 (12)	−0.0040 (11)	0.0042 (10)	−0.0005 (11)
C27B	0.0247 (13)	0.0301 (16)	0.0227 (14)	−0.0006 (12)	0.0009 (12)	−0.0001 (13)
C28B	0.0222 (13)	0.0321 (17)	0.0206 (13)	−0.0045 (12)	0.0005 (11)	−0.0001 (13)
C29B	0.0386 (17)	0.043 (2)	0.0295 (17)	0.0080 (16)	−0.0087 (14)	−0.0026 (16)
C30B	0.0268 (14)	0.048 (2)	0.0270 (15)	0.0015 (14)	−0.0016 (13)	−0.0019 (15)
C31B	0.011 (3)	0.055 (4)	0.043 (4)	0.000 (3)	0.003 (2)	−0.005 (3)
C32B	0.029 (3)	0.047 (3)	0.048 (4)	0.001 (3)	−0.010 (3)	0.004 (3)
C31Y	0.019 (5)	0.121 (11)	0.037 (5)	0.010 (6)	0.015 (4)	0.007 (7)
C32Y	0.029 (6)	0.071 (6)	0.025 (5)	0.010 (5)	−0.009 (4)	−0.007 (4)

### Geometric parameters (Å, °)

**Table:**

O1A—C21A	1.334 (4)	C31X—H31E	0.9600
O1A—C5A	1.457 (3)	C31X—H31F	0.9600
O2A—C21A	1.209 (4)	C32X—H32D	0.9600
O3A—C18A	1.228 (3)	C32X—H32E	0.9600
N1A—C2A	1.287 (3)	C32X—H32F	0.9600
N1A—N2A	1.394 (3)	O1B—C21B	1.353 (4)
N2A—C18A	1.352 (3)	O1B—C5B	1.460 (3)
N2A—H1NA	0.85 (3)	O2B—C21B	1.199 (4)
N3A—C20A	1.143 (3)	O3B—C18B	1.225 (3)
C1A—C2A	1.506 (3)	N1B—C2B	1.283 (3)
C1A—C17A	1.534 (3)	N1B—N2B	1.389 (3)
C1A—H1AA	0.9700	N2B—C18B	1.344 (3)
C1A—H1AB	0.9700	N2B—H1NB	0.88 (3)
C2A—C3A	1.516 (3)	N3B—C20B	1.147 (4)
C3A—C4A	1.533 (3)	C1B—C2B	1.508 (3)
C3A—C8A	1.554 (4)	C1B—C17B	1.540 (4)
C3A—H3AA	0.9800	C1B—H1BA	0.9700
C4A—C5A	1.524 (3)	C1B—H1BB	0.9700
C4A—H4AA	0.9700	C2B—C3B	1.504 (3)
C4A—H4AB	0.9700	C3B—C4B	1.534 (3)
C5A—C6A	1.506 (4)	C3B—C8B	1.566 (4)
C5A—H5AA	0.9800	C3B—H3BA	0.9800
C6A—C7A	1.540 (3)	C4B—C5B	1.523 (3)
C6A—H6AA	0.9700	C4B—H4BA	0.9700
C6A—H6AB	0.9700	C4B—H4BB	0.9700
C7A—C8A	1.537 (3)	C5B—C6B	1.512 (4)
C7A—H7AA	0.9700	C5B—H5BA	0.9800
C7A—H7AB	0.9700	C6B—C7B	1.533 (3)
C8A—C23A	1.532 (4)	C6B—H6BA	0.9700
C8A—C9A	1.555 (3)	C6B—H6BB	0.9700
C9A—C10A	1.534 (3)	C7B—C8B	1.543 (3)
C9A—C17A	1.539 (3)	C7B—H7BA	0.9700
C9A—H9AA	0.9800	C7B—H7BB	0.9700
C10A—C11A	1.539 (3)	C8B—C23B	1.542 (4)
C10A—H10A	0.9700	C8B—C9B	1.553 (3)
C10A—H10B	0.9700	C9B—C10B	1.530 (4)
C11A—C12A	1.531 (3)	C9B—C17B	1.546 (3)
C11A—H11A	0.9700	C9B—H9BA	0.9800
C11A—H11B	0.9700	C10B—C11B	1.532 (4)
C12A—C24A	1.534 (4)	C10B—H10C	0.9700
C12A—C16A	1.543 (3)	C10B—H10D	0.9700
C12A—C13A	1.562 (3)	C11B—C12B	1.535 (3)
C13A—C26A	1.540 (4)	C11B—H11C	0.9700
C13A—C14A	1.556 (3)	C11B—H11D	0.9700
C13A—H13A	0.9800	C12B—C24B	1.536 (4)
C14A—C15A	1.548 (3)	C12B—C13B	1.550 (3)
C14A—H14A	0.9700	C12B—C16B	1.552 (4)
C14A—H14B	0.9700	C13B—C26B	1.544 (3)
C15A—C16A	1.533 (3)	C13B—C14B	1.558 (4)
C15A—H15A	0.9700	C13B—H13B	0.9800
C15A—H15B	0.9700	C14B—C15B	1.550 (4)
C16A—C17A	1.529 (3)	C14B—H14C	0.9700
C16A—H16A	0.9800	C14B—H14D	0.9700
C17A—H17A	0.9800	C15B—C16B	1.523 (3)
C18A—C19A	1.524 (3)	C15B—H15C	0.9700
C19A—C20A	1.469 (4)	C15B—H15D	0.9700
C19A—H19A	0.9700	C16B—C17B	1.529 (3)
C19A—H19B	0.9700	C16B—H16B	0.9800
C21A—C22A	1.499 (4)	C17B—H17B	0.9800
C22A—H22A	0.9600	C18B—C19B	1.531 (3)
C22A—H22B	0.9600	C19B—C20B	1.470 (4)
C22A—H22C	0.9600	C19B—H19C	0.9700
C23A—H23A	0.9600	C19B—H19D	0.9700
C23A—H23B	0.9600	C21B—C22B	1.506 (4)
C23A—H23C	0.9600	C22B—H22D	0.9600
C24A—H24A	0.9600	C22B—H22E	0.9600
C24A—H24B	0.9600	C22B—H22F	0.9600
C24A—H24C	0.9600	C23B—H23D	0.9600
C25A—C26A	1.537 (4)	C23B—H23E	0.9600
C25A—H25A	0.9600	C23B—H23F	0.9600
C25A—H25B	0.9600	C24B—H24D	0.9600
C25A—H25C	0.9600	C24B—H24E	0.9600
C26A—C27X	1.31 (2)	C24B—H24F	0.9600
C26A—C27A	1.587 (5)	C25B—C26B	1.530 (4)
C26A—H26B	0.9800	C25B—H25D	0.9600
C26A—H26C	0.9800	C25B—H25E	0.9600
C27A—C28A	1.532 (5)	C25B—H25F	0.9600
C27A—H27A	0.9700	C26B—C27B	1.530 (4)
C27A—H27B	0.9700	C26B—H26A	0.9800
C28A—C29A	1.530 (5)	C27B—C28B	1.529 (4)
C28A—H28A	0.9700	C27B—H27E	0.9700
C28A—H28B	0.9700	C27B—H27F	0.9700
C29A—C30A	1.516 (6)	C28B—C29B	1.516 (4)
C29A—H29A	0.9700	C28B—H28E	0.9700
C29A—H29B	0.9700	C28B—H28F	0.9700
C30A—C32A	1.530 (5)	C29B—C30B	1.524 (4)
C30A—C31A	1.534 (5)	C29B—H29D	0.9700
C30A—H30A	0.9800	C29B—H29E	0.9700
C31A—H31A	0.9600	C30B—C31Y	1.480 (8)
C31A—H31B	0.9600	C30B—C31B	1.542 (8)
C31A—H31C	0.9600	C30B—C32Y	1.543 (8)
C32A—H32A	0.9600	C30B—C32B	1.549 (7)
C32A—H32B	0.9600	C30B—H30C	0.9800
C32A—H32C	0.9600	C30B—H30D	0.9800
C27X—C28X	1.52 (3)	C31B—H31G	0.9600
C27X—H27C	0.9700	C31B—H31H	0.9600
C27X—H27D	0.9700	C31B—H31I	0.9600
C28X—C29X	1.56 (2)	C32B—H32G	0.9600
C28X—H28C	0.9700	C32B—H32H	0.9600
C28X—H28D	0.9700	C32B—H32I	0.9600
C29X—C30X	1.50 (3)	C31Y—H31J	0.9600
C29X—H29C	0.9700	C31Y—H31K	0.9600
C29X—H29F	0.9700	C31Y—H31L	0.9600
C30X—C32X	1.534 (10)	C32Y—H32J	0.9600
C30X—C31X	1.535 (10)	C32Y—H32K	0.9600
C30X—H30B	0.9800	C32Y—H32L	0.9600
C31X—H31D	0.9600		
			
C21A—O1A—C5A	117.7 (2)	C30X—C31X—H31F	109.5
C2A—N1A—N2A	118.00 (19)	H31D—C31X—H31F	109.5
C18A—N2A—N1A	120.3 (2)	H31E—C31X—H31F	109.5
C18A—N2A—H1NA	117 (2)	C30X—C32X—H32D	109.5
N1A—N2A—H1NA	122 (2)	C30X—C32X—H32E	109.5
C2A—C1A—C17A	115.6 (2)	H32D—C32X—H32E	109.5
C2A—C1A—H1AA	108.4	C30X—C32X—H32F	109.5
C17A—C1A—H1AA	108.4	H32D—C32X—H32F	109.5
C2A—C1A—H1AB	108.4	H32E—C32X—H32F	109.5
C17A—C1A—H1AB	108.4	C21B—O1B—C5B	116.0 (2)
H1AA—C1A—H1AB	107.4	C2B—N1B—N2B	118.8 (2)
N1A—C2A—C1A	126.3 (2)	C18B—N2B—N1B	117.2 (2)
N1A—C2A—C3A	116.4 (2)	C18B—N2B—H1NB	117.1 (19)
C1A—C2A—C3A	117.0 (2)	N1B—N2B—H1NB	123.1 (18)
C2A—C3A—C4A	114.2 (2)	C2B—C1B—C17B	110.2 (2)
C2A—C3A—C8A	111.7 (2)	C2B—C1B—H1BA	109.6
C4A—C3A—C8A	113.2 (2)	C17B—C1B—H1BA	109.6
C2A—C3A—H3AA	105.6	C2B—C1B—H1BB	109.6
C4A—C3A—H3AA	105.6	C17B—C1B—H1BB	109.6
C8A—C3A—H3AA	105.6	H1BA—C1B—H1BB	108.1
C5A—C4A—C3A	110.0 (2)	N1B—C2B—C3B	116.1 (2)
C5A—C4A—H4AA	109.7	N1B—C2B—C1B	129.2 (2)
C3A—C4A—H4AA	109.7	C3B—C2B—C1B	114.1 (2)
C5A—C4A—H4AB	109.7	C2B—C3B—C4B	115.2 (2)
C3A—C4A—H4AB	109.7	C2B—C3B—C8B	106.25 (19)
H4AA—C4A—H4AB	108.2	C4B—C3B—C8B	113.4 (2)
O1A—C5A—C6A	105.8 (2)	C2B—C3B—H3BA	107.2
O1A—C5A—C4A	109.8 (2)	C4B—C3B—H3BA	107.2
C6A—C5A—C4A	112.4 (2)	C8B—C3B—H3BA	107.2
O1A—C5A—H5AA	109.6	C5B—C4B—C3B	108.2 (2)
C6A—C5A—H5AA	109.6	C5B—C4B—H4BA	110.1
C4A—C5A—H5AA	109.6	C3B—C4B—H4BA	110.1
C5A—C6A—C7A	110.8 (2)	C5B—C4B—H4BB	110.1
C5A—C6A—H6AA	109.5	C3B—C4B—H4BB	110.1
C7A—C6A—H6AA	109.5	H4BA—C4B—H4BB	108.4
C5A—C6A—H6AB	109.5	O1B—C5B—C6B	107.1 (2)
C7A—C6A—H6AB	109.5	O1B—C5B—C4B	111.1 (2)
H6AA—C6A—H6AB	108.1	C6B—C5B—C4B	110.6 (2)
C8A—C7A—C6A	113.9 (2)	O1B—C5B—H5BA	109.3
C8A—C7A—H7AA	108.8	C6B—C5B—H5BA	109.3
C6A—C7A—H7AA	108.8	C4B—C5B—H5BA	109.3
C8A—C7A—H7AB	108.8	C5B—C6B—C7B	108.9 (2)
C6A—C7A—H7AB	108.8	C5B—C6B—H6BA	109.9
H7AA—C7A—H7AB	107.7	C7B—C6B—H6BA	109.9
C23A—C8A—C7A	110.1 (2)	C5B—C6B—H6BB	109.9
C23A—C8A—C3A	110.5 (2)	C7B—C6B—H6BB	109.9
C7A—C8A—C3A	108.3 (2)	H6BA—C6B—H6BB	108.3
C23A—C8A—C9A	110.9 (2)	C6B—C7B—C8B	113.9 (2)
C7A—C8A—C9A	109.6 (2)	C6B—C7B—H7BA	108.8
C3A—C8A—C9A	107.42 (19)	C8B—C7B—H7BA	108.8
C10A—C9A—C17A	111.78 (19)	C6B—C7B—H7BB	108.8
C10A—C9A—C8A	114.92 (19)	C8B—C7B—H7BB	108.8
C17A—C9A—C8A	111.30 (19)	H7BA—C7B—H7BB	107.7
C10A—C9A—H9AA	106.0	C23B—C8B—C7B	108.9 (2)
C17A—C9A—H9AA	106.0	C23B—C8B—C9B	111.5 (2)
C8A—C9A—H9AA	106.0	C7B—C8B—C9B	109.72 (19)
C9A—C10A—C11A	112.8 (2)	C23B—C8B—C3B	111.31 (19)
C9A—C10A—H10A	109.0	C7B—C8B—C3B	108.70 (19)
C11A—C10A—H10A	109.0	C9B—C8B—C3B	106.66 (19)
C9A—C10A—H10B	109.0	C10B—C9B—C17B	109.3 (2)
C11A—C10A—H10B	109.0	C10B—C9B—C8B	114.3 (2)
H10A—C10A—H10B	107.8	C17B—C9B—C8B	113.71 (19)
C12A—C11A—C10A	112.2 (2)	C10B—C9B—H9BA	106.3
C12A—C11A—H11A	109.2	C17B—C9B—H9BA	106.3
C10A—C11A—H11A	109.2	C8B—C9B—H9BA	106.3
C12A—C11A—H11B	109.2	C9B—C10B—C11B	113.4 (2)
C10A—C11A—H11B	109.2	C9B—C10B—H10C	108.9
H11A—C11A—H11B	107.9	C11B—C10B—H10C	108.9
C11A—C12A—C24A	110.4 (2)	C9B—C10B—H10D	108.9
C11A—C12A—C16A	107.0 (2)	C11B—C10B—H10D	108.9
C24A—C12A—C16A	112.3 (2)	H10C—C10B—H10D	107.7
C11A—C12A—C13A	117.3 (2)	C10B—C11B—C12B	112.3 (2)
C24A—C12A—C13A	109.7 (2)	C10B—C11B—H11C	109.1
C16A—C12A—C13A	99.55 (19)	C12B—C11B—H11C	109.1
C26A—C13A—C14A	112.0 (2)	C10B—C11B—H11D	109.1
C26A—C13A—C12A	119.6 (2)	C12B—C11B—H11D	109.1
C14A—C13A—C12A	102.96 (19)	H11C—C11B—H11D	107.9
C26A—C13A—H13A	107.2	C11B—C12B—C24B	110.9 (2)
C14A—C13A—H13A	107.2	C11B—C12B—C13B	116.5 (2)
C12A—C13A—H13A	107.2	C24B—C12B—C13B	110.5 (2)
C15A—C14A—C13A	107.14 (18)	C11B—C12B—C16B	107.02 (19)
C15A—C14A—H14A	110.3	C24B—C12B—C16B	112.0 (2)
C13A—C14A—H14A	110.3	C13B—C12B—C16B	99.36 (19)
C15A—C14A—H14B	110.3	C26B—C13B—C12B	120.3 (2)
C13A—C14A—H14B	110.3	C26B—C13B—C14B	112.5 (2)
H14A—C14A—H14B	108.5	C12B—C13B—C14B	103.64 (19)
C16A—C15A—C14A	103.45 (19)	C26B—C13B—H13B	106.5
C16A—C15A—H15A	111.1	C12B—C13B—H13B	106.5
C14A—C15A—H15A	111.1	C14B—C13B—H13B	106.5
C16A—C15A—H15B	111.1	C15B—C14B—C13B	106.8 (2)
C14A—C15A—H15B	111.1	C15B—C14B—H14C	110.4
H15A—C15A—H15B	109.0	C13B—C14B—H14C	110.4
C17A—C16A—C15A	118.5 (2)	C15B—C14B—H14D	110.4
C17A—C16A—C12A	114.6 (2)	C13B—C14B—H14D	110.4
C15A—C16A—C12A	104.39 (19)	H14C—C14B—H14D	108.6
C17A—C16A—H16A	106.2	C16B—C15B—C14B	103.5 (2)
C15A—C16A—H16A	106.2	C16B—C15B—H15C	111.1
C12A—C16A—H16A	106.2	C14B—C15B—H15C	111.1
C16A—C17A—C1A	110.6 (2)	C16B—C15B—H15D	111.1
C16A—C17A—C9A	109.15 (19)	C14B—C15B—H15D	111.1
C1A—C17A—C9A	111.6 (2)	H15C—C15B—H15D	109.0
C16A—C17A—H17A	108.5	C15B—C16B—C17B	119.7 (2)
C1A—C17A—H17A	108.5	C15B—C16B—C12B	104.58 (19)
C9A—C17A—H17A	108.5	C17B—C16B—C12B	114.0 (2)
O3A—C18A—N2A	121.0 (2)	C15B—C16B—H16B	105.9
O3A—C18A—C19A	121.8 (2)	C17B—C16B—H16B	105.9
N2A—C18A—C19A	117.2 (2)	C12B—C16B—H16B	105.9
C20A—C19A—C18A	110.7 (2)	C16B—C17B—C1B	112.1 (2)
C20A—C19A—H19A	109.5	C16B—C17B—C9B	107.37 (19)
C18A—C19A—H19A	109.5	C1B—C17B—C9B	112.54 (19)
C20A—C19A—H19B	109.5	C16B—C17B—H17B	108.2
C18A—C19A—H19B	109.5	C1B—C17B—H17B	108.2
H19A—C19A—H19B	108.1	C9B—C17B—H17B	108.2
N3A—C20A—C19A	177.9 (3)	O3B—C18B—N2B	122.6 (2)
O2A—C21A—O1A	123.7 (3)	O3B—C18B—C19B	120.6 (2)
O2A—C21A—C22A	125.3 (3)	N2B—C18B—C19B	116.7 (2)
O1A—C21A—C22A	111.0 (3)	C20B—C19B—C18B	112.6 (2)
C21A—C22A—H22A	109.5	C20B—C19B—H19C	109.1
C21A—C22A—H22B	109.5	C18B—C19B—H19C	109.1
H22A—C22A—H22B	109.5	C20B—C19B—H19D	109.1
C21A—C22A—H22C	109.5	C18B—C19B—H19D	109.1
H22A—C22A—H22C	109.5	H19C—C19B—H19D	107.8
H22B—C22A—H22C	109.5	N3B—C20B—C19B	178.5 (3)
C8A—C23A—H23A	109.5	O2B—C21B—O1B	124.1 (3)
C8A—C23A—H23B	109.5	O2B—C21B—C22B	125.3 (3)
H23A—C23A—H23B	109.5	O1B—C21B—C22B	110.7 (3)
C8A—C23A—H23C	109.5	C21B—C22B—H22D	109.5
H23A—C23A—H23C	109.5	C21B—C22B—H22E	109.5
H23B—C23A—H23C	109.5	H22D—C22B—H22E	109.5
C12A—C24A—H24A	109.5	C21B—C22B—H22F	109.5
C12A—C24A—H24B	109.5	H22D—C22B—H22F	109.5
H24A—C24A—H24B	109.5	H22E—C22B—H22F	109.5
C12A—C24A—H24C	109.5	C8B—C23B—H23D	109.5
H24A—C24A—H24C	109.5	C8B—C23B—H23E	109.5
H24B—C24A—H24C	109.5	H23D—C23B—H23E	109.5
C26A—C25A—H25A	109.5	C8B—C23B—H23F	109.5
C26A—C25A—H25B	109.5	H23D—C23B—H23F	109.5
H25A—C25A—H25B	109.5	H23E—C23B—H23F	109.5
C26A—C25A—H25C	109.5	C12B—C24B—H24D	109.5
H25A—C25A—H25C	109.5	C12B—C24B—H24E	109.5
H25B—C25A—H25C	109.5	H24D—C24B—H24E	109.5
C27X—C26A—C25A	112.8 (9)	C12B—C24B—H24F	109.5
C27X—C26A—C13A	122.6 (10)	H24D—C24B—H24F	109.5
C25A—C26A—C13A	113.1 (2)	H24E—C24B—H24F	109.5
C25A—C26A—C27A	108.9 (2)	C26B—C25B—H25D	109.5
C13A—C26A—C27A	109.1 (3)	C26B—C25B—H25E	109.5
C27X—C26A—H26B	87.3	H25D—C25B—H25E	109.5
C25A—C26A—H26B	108.5	C26B—C25B—H25F	109.5
C13A—C26A—H26B	108.5	H25D—C25B—H25F	109.5
C27A—C26A—H26B	108.5	H25E—C25B—H25F	109.5
C27X—C26A—H26C	101.4	C25B—C26B—C27B	110.2 (2)
C25A—C26A—H26C	101.4	C25B—C26B—C13B	112.6 (2)
C13A—C26A—H26C	101.4	C27B—C26B—C13B	109.4 (2)
C27A—C26A—H26C	122.7	C25B—C26B—H26A	108.2
C28A—C27A—C26A	113.1 (3)	C27B—C26B—H26A	108.2
C28A—C27A—H27A	109.0	C13B—C26B—H26A	108.2
C26A—C27A—H27A	109.0	C28B—C27B—C26B	116.6 (2)
C28A—C27A—H27B	109.0	C28B—C27B—H27E	108.1
C26A—C27A—H27B	109.0	C26B—C27B—H27E	108.1
H27A—C27A—H27B	107.8	C28B—C27B—H27F	108.1
C29A—C28A—C27A	114.9 (3)	C26B—C27B—H27F	108.1
C29A—C28A—H28A	108.5	H27E—C27B—H27F	107.3
C27A—C28A—H28A	108.5	C29B—C28B—C27B	113.0 (3)
C29A—C28A—H28B	108.5	C29B—C28B—H28E	109.0
C27A—C28A—H28B	108.5	C27B—C28B—H28E	109.0
H28A—C28A—H28B	107.5	C29B—C28B—H28F	109.0
C30A—C29A—C28A	116.2 (3)	C27B—C28B—H28F	109.0
C30A—C29A—H29A	108.2	H28E—C28B—H28F	107.8
C28A—C29A—H29A	108.2	C28B—C29B—C30B	116.0 (3)
C30A—C29A—H29B	108.2	C28B—C29B—H29D	108.3
C28A—C29A—H29B	108.2	C30B—C29B—H29D	108.3
H29A—C29A—H29B	107.4	C28B—C29B—H29E	108.3
C29A—C30A—C32A	109.9 (3)	C30B—C29B—H29E	108.3
C29A—C30A—C31A	112.1 (4)	H29D—C29B—H29E	107.4
C32A—C30A—C31A	109.5 (3)	C31Y—C30B—C29B	108.3 (7)
C29A—C30A—H30A	108.4	C29B—C30B—C31B	112.1 (4)
C32A—C30A—H30A	108.4	C31Y—C30B—C32Y	114.4 (7)
C31A—C30A—H30A	108.4	C29B—C30B—C32Y	110.5 (5)
C26A—C27X—C28X	108.6 (16)	C29B—C30B—C32B	110.8 (4)
C26A—C27X—H27C	110.0	C31B—C30B—C32B	106.6 (4)
C28X—C27X—H27C	110.0	C29B—C30B—H30C	109.1
C26A—C27X—H27D	110.0	C31B—C30B—H30C	109.1
C28X—C27X—H27D	110.0	C32B—C30B—H30C	109.1
H27C—C27X—H27D	108.4	C31Y—C30B—H30D	107.8
C27X—C28X—C29X	112.0 (16)	C29B—C30B—H30D	107.8
C27X—C28X—H28C	109.2	C32Y—C30B—H30D	107.8
C29X—C28X—H28C	109.2	C30B—C31B—H31G	109.5
C27X—C28X—H28D	109.2	C30B—C31B—H31H	109.5
C29X—C28X—H28D	109.2	C30B—C31B—H31I	109.5
H28C—C28X—H28D	107.9	C30B—C32B—H32G	109.5
C30X—C29X—C28X	115.4 (14)	C30B—C32B—H32H	109.5
C30X—C29X—H29C	108.4	C30B—C32B—H32I	109.5
C28X—C29X—H29C	108.4	C30B—C31Y—H31J	109.5
C30X—C29X—H29F	108.4	C30B—C31Y—H31K	109.5
C28X—C29X—H29F	108.4	H31J—C31Y—H31K	109.5
H29C—C29X—H29F	107.5	C30B—C31Y—H31L	109.5
C29X—C30X—C32X	110.3 (17)	H31J—C31Y—H31L	109.5
C29X—C30X—C31X	103.9 (19)	H31K—C31Y—H31L	109.5
C32X—C30X—C31X	105.8 (18)	C30B—C32Y—H32J	109.5
C29X—C30X—H30B	112.1	C30B—C32Y—H32K	109.5
C32X—C30X—H30B	112.1	H32J—C32Y—H32K	109.5
C31X—C30X—H30B	112.1	C30B—C32Y—H32L	109.5
C30X—C31X—H31D	109.5	H32J—C32Y—H32L	109.5
C30X—C31X—H31E	109.5	H32K—C32Y—H32L	109.5
H31D—C31X—H31E	109.5		
			
C2A—N1A—N2A—C18A	−173.8 (2)	C26A—C27X—C28X—C29X	174.8 (14)
N2A—N1A—C2A—C1A	−0.4 (4)	C27X—C28X—C29X—C30X	−69 (2)
N2A—N1A—C2A—C3A	173.6 (2)	C28X—C29X—C30X—C32X	163.5 (17)
C17A—C1A—C2A—N1A	−147.5 (3)	C28X—C29X—C30X—C31X	−83 (2)
C17A—C1A—C2A—C3A	38.5 (3)	C2B—N1B—N2B—C18B	174.0 (2)
N1A—C2A—C3A—C4A	8.8 (3)	N2B—N1B—C2B—C3B	−168.8 (2)
C1A—C2A—C3A—C4A	−176.6 (2)	N2B—N1B—C2B—C1B	1.5 (4)
N1A—C2A—C3A—C8A	138.9 (2)	C17B—C1B—C2B—N1B	−113.6 (3)
C1A—C2A—C3A—C8A	−46.5 (3)	C17B—C1B—C2B—C3B	56.9 (3)
C2A—C3A—C4A—C5A	−174.4 (2)	N1B—C2B—C3B—C4B	−20.9 (3)
C8A—C3A—C4A—C5A	56.3 (3)	C1B—C2B—C3B—C4B	167.3 (2)
C21A—O1A—C5A—C6A	142.9 (2)	N1B—C2B—C3B—C8B	105.6 (2)
C21A—O1A—C5A—C4A	−95.6 (3)	C1B—C2B—C3B—C8B	−66.2 (3)
C3A—C4A—C5A—O1A	−173.8 (2)	C2B—C3B—C4B—C5B	−179.9 (2)
C3A—C4A—C5A—C6A	−56.3 (3)	C8B—C3B—C4B—C5B	57.4 (3)
O1A—C5A—C6A—C7A	175.0 (2)	C21B—O1B—C5B—C6B	154.0 (2)
C4A—C5A—C6A—C7A	55.2 (3)	C21B—O1B—C5B—C4B	−85.2 (3)
C5A—C6A—C7A—C8A	−54.5 (3)	C3B—C4B—C5B—O1B	178.8 (2)
C6A—C7A—C8A—C23A	−68.2 (3)	C3B—C4B—C5B—C6B	−62.4 (3)
C6A—C7A—C8A—C3A	52.7 (3)	O1B—C5B—C6B—C7B	−176.7 (2)
C6A—C7A—C8A—C9A	169.6 (2)	C4B—C5B—C6B—C7B	62.1 (3)
C2A—C3A—C8A—C23A	−63.9 (3)	C5B—C6B—C7B—C8B	−56.9 (3)
C4A—C3A—C8A—C23A	66.7 (3)	C6B—C7B—C8B—C23B	−70.8 (3)
C2A—C3A—C8A—C7A	175.4 (2)	C6B—C7B—C8B—C9B	166.9 (2)
C4A—C3A—C8A—C7A	−54.0 (3)	C6B—C7B—C8B—C3B	50.6 (3)
C2A—C3A—C8A—C9A	57.2 (3)	C2B—C3B—C8B—C23B	−58.8 (2)
C4A—C3A—C8A—C9A	−172.2 (2)	C4B—C3B—C8B—C23B	68.8 (3)
C23A—C8A—C9A—C10A	−70.4 (3)	C2B—C3B—C8B—C7B	−178.74 (19)
C7A—C8A—C9A—C10A	51.3 (3)	C4B—C3B—C8B—C7B	−51.2 (3)
C3A—C8A—C9A—C10A	168.7 (2)	C2B—C3B—C8B—C9B	63.0 (2)
C23A—C8A—C9A—C17A	57.9 (3)	C4B—C3B—C8B—C9B	−169.40 (19)
C7A—C8A—C9A—C17A	179.6 (2)	C23B—C8B—C9B—C10B	−62.1 (3)
C3A—C8A—C9A—C17A	−63.0 (3)	C7B—C8B—C9B—C10B	58.6 (3)
C17A—C9A—C10A—C11A	52.4 (3)	C3B—C8B—C9B—C10B	176.2 (2)
C8A—C9A—C10A—C11A	−179.5 (2)	C23B—C8B—C9B—C17B	64.4 (3)
C9A—C10A—C11A—C12A	−55.0 (3)	C7B—C8B—C9B—C17B	−174.9 (2)
C10A—C11A—C12A—C24A	−67.1 (3)	C3B—C8B—C9B—C17B	−57.3 (3)
C10A—C11A—C12A—C16A	55.5 (3)	C17B—C9B—C10B—C11B	56.4 (3)
C10A—C11A—C12A—C13A	166.2 (2)	C8B—C9B—C10B—C11B	−174.8 (2)
C11A—C12A—C13A—C26A	78.7 (3)	C9B—C10B—C11B—C12B	−54.9 (3)
C24A—C12A—C13A—C26A	−48.4 (3)	C10B—C11B—C12B—C24B	−69.8 (3)
C16A—C12A—C13A—C26A	−166.4 (2)	C10B—C11B—C12B—C13B	162.7 (2)
C11A—C12A—C13A—C14A	−156.4 (2)	C10B—C11B—C12B—C16B	52.7 (3)
C24A—C12A—C13A—C14A	76.6 (2)	C11B—C12B—C13B—C26B	78.3 (3)
C16A—C12A—C13A—C14A	−41.5 (2)	C24B—C12B—C13B—C26B	−49.5 (3)
C26A—C13A—C14A—C15A	151.6 (2)	C16B—C12B—C13B—C26B	−167.3 (2)
C12A—C13A—C14A—C15A	21.8 (3)	C11B—C12B—C13B—C14B	−155.0 (2)
C13A—C14A—C15A—C16A	7.0 (3)	C24B—C12B—C13B—C14B	77.3 (2)
C14A—C15A—C16A—C17A	−162.9 (2)	C16B—C12B—C13B—C14B	−40.6 (2)
C14A—C15A—C16A—C12A	−34.0 (3)	C26B—C13B—C14B—C15B	152.4 (2)
C11A—C12A—C16A—C17A	−58.9 (3)	C12B—C13B—C14B—C15B	20.9 (3)
C24A—C12A—C16A—C17A	62.4 (3)	C13B—C14B—C15B—C16B	8.1 (3)
C13A—C12A—C16A—C17A	178.5 (2)	C14B—C15B—C16B—C17B	−163.6 (2)
C11A—C12A—C16A—C15A	169.9 (2)	C14B—C15B—C16B—C12B	−34.4 (3)
C24A—C12A—C16A—C15A	−68.8 (3)	C11B—C12B—C16B—C15B	168.6 (2)
C13A—C12A—C16A—C15A	47.3 (2)	C24B—C12B—C16B—C15B	−69.6 (3)
C15A—C16A—C17A—C1A	−55.1 (3)	C13B—C12B—C16B—C15B	47.1 (2)
C12A—C16A—C17A—C1A	−179.1 (2)	C11B—C12B—C16B—C17B	−58.9 (3)
C15A—C16A—C17A—C9A	−178.2 (2)	C24B—C12B—C16B—C17B	62.9 (3)
C12A—C16A—C17A—C9A	57.7 (3)	C13B—C12B—C16B—C17B	179.58 (19)
C2A—C1A—C17A—C16A	−163.5 (2)	C15B—C16B—C17B—C1B	−48.9 (3)
C2A—C1A—C17A—C9A	−41.8 (3)	C12B—C16B—C17B—C1B	−173.71 (19)
C10A—C9A—C17A—C16A	−52.2 (3)	C15B—C16B—C17B—C9B	−173.0 (2)
C8A—C9A—C17A—C16A	177.8 (2)	C12B—C16B—C17B—C9B	62.2 (3)
C10A—C9A—C17A—C1A	−174.7 (2)	C2B—C1B—C17B—C16B	−166.9 (2)
C8A—C9A—C17A—C1A	55.3 (3)	C2B—C1B—C17B—C9B	−45.7 (3)
N1A—N2A—C18A—O3A	174.5 (2)	C10B—C9B—C17B—C16B	−57.9 (3)
N1A—N2A—C18A—C19A	−4.8 (4)	C8B—C9B—C17B—C16B	173.0 (2)
O3A—C18A—C19A—C20A	36.2 (4)	C10B—C9B—C17B—C1B	178.3 (2)
N2A—C18A—C19A—C20A	−144.5 (3)	C8B—C9B—C17B—C1B	49.2 (3)
C5A—O1A—C21A—O2A	3.5 (4)	N1B—N2B—C18B—O3B	−173.1 (2)
C5A—O1A—C21A—C22A	−179.1 (2)	N1B—N2B—C18B—C19B	8.4 (3)
C14A—C13A—C26A—C27X	46.7 (12)	O3B—C18B—C19B—C20B	113.1 (3)
C12A—C13A—C26A—C27X	167.2 (12)	N2B—C18B—C19B—C20B	−68.3 (3)
C14A—C13A—C26A—C25A	−172.7 (2)	C5B—O1B—C21B—O2B	7.5 (4)
C12A—C13A—C26A—C25A	−52.2 (3)	C5B—O1B—C21B—C22B	−171.9 (2)
C14A—C13A—C26A—C27A	65.9 (3)	C12B—C13B—C26B—C25B	−48.8 (3)
C12A—C13A—C26A—C27A	−173.6 (2)	C14B—C13B—C26B—C25B	−171.4 (2)
C27X—C26A—C27A—C28A	−35 (3)	C12B—C13B—C26B—C27B	−171.7 (2)
C25A—C26A—C27A—C28A	69.0 (4)	C14B—C13B—C26B—C27B	65.7 (3)
C13A—C26A—C27A—C28A	−167.2 (3)	C25B—C26B—C27B—C28B	58.5 (3)
C26A—C27A—C28A—C29A	−179.5 (3)	C13B—C26B—C27B—C28B	−177.2 (2)
C27A—C28A—C29A—C30A	60.4 (5)	C26B—C27B—C28B—C29B	179.8 (3)
C28A—C29A—C30A—C32A	173.3 (3)	C27B—C28B—C29B—C30B	167.3 (3)
C28A—C29A—C30A—C31A	51.3 (4)	C28B—C29B—C30B—C31Y	75.6 (10)
C25A—C26A—C27X—C28X	−74.9 (16)	C28B—C29B—C30B—C31B	51.0 (5)
C13A—C26A—C27X—C28X	65.8 (17)	C28B—C29B—C30B—C32Y	−158.5 (8)
C27A—C26A—C27X—C28X	9.2 (16)	C28B—C29B—C30B—C32B	170.0 (6)

### Hydrogen-bond geometry (Å, °)

**Table:**

*D*—H···*A*	*D*—H	H···*A*	*D*···*A*	*D*—H···*A*
N2A—H1NA···O3B	0.85 (3)	2.09 (3)	2.932 (3)	170 (3)
N2B—H1NB···O3A	0.88 (3)	2.11 (3)	2.963 (3)	163 (3)
C1A—H1AB···O3B	0.97	2.39	3.201 (3)	140
